# Histological and global gene expression analysis of the 'lactating' pigeon crop

**DOI:** 10.1186/1471-2164-12-452

**Published:** 2011-09-19

**Authors:** Meagan J Gillespie, Volker R Haring, Kenneth A McColl, Paul Monaghan, John A Donald, Kevin R Nicholas, Robert J Moore, Tamsyn M Crowley

**Affiliations:** 1 CSIRO Livestock Industries, Australian Animal Health Laboratory, 5 Portarlington Road, Geelong, VIC, Australia; 2 School of Life and Environmental Sciences, Deakin University, Pigdons Road, Geelong, VIC, Australia; 3 Institute for Technology Research and Innovation, Deakin University, Pigdons Road, Geelong, VIC, Australia

## Abstract

**Background:**

Both male and female pigeons have the ability to produce a nutrient solution in their crop for the nourishment of their young. The production of the nutrient solution has been likened to lactation in mammals, and hence the product has been called pigeon 'milk'. It has been shown that pigeon 'milk' is essential for growth and development of the pigeon squab, and without it they fail to thrive. Studies have investigated the nutritional value of pigeon 'milk' but very little else is known about what it is or how it is produced. This study aimed to gain insight into the process by studying gene expression in the 'lactating' crop.

**Results:**

Macroscopic comparison of 'lactating' and non-'lactating' crop reveals that the 'lactating' crop is enlarged and thickened with two very obvious lateral lobes that contain discrete rice-shaped pellets of pigeon 'milk'. This was characterised histologically by an increase in the number and depth of rete pegs extending from the basal layer of the epithelium to the lamina propria, and extensive proliferation and folding of the germinal layer into the superficial epithelium. A global gene expression profile comparison between 'lactating' crop and non-'lactating' crop showed that 542 genes are up-regulated in the 'lactating' crop, and 639 genes are down-regulated. Pathway analysis revealed that genes up-regulated in 'lactating' crop were involved in the proliferation of melanocytes, extracellular matrix-receptor interaction, the adherens junction and the wingless (wnt) signalling pathway. Gene ontology analysis showed that antioxidant response and microtubule transport were enriched in 'lactating' crop.

**Conclusions:**

There is a hyperplastic response in the pigeon crop epithelium during 'lactation' that leads to localised cellular stress and expression of antioxidant protein-encoding genes. The differentiated, cornified cells that form the pigeon 'milk' are of keratinocyte lineage and contain triglycerides that are likely endocytosed as very low density lipoprotein (VLDL) and repackaged as triglyceride in vesicles that are transported intracellularly by microtubules. This mechanism is an interesting example of the evolution of a system with analogies to mammalian lactation, as pigeon 'milk' fulfils a similar function to mammalian milk, but is produced by a different mechanism.

## Background

Both male and female pigeons (*Columba livia*) possess the ability to produce a complete nutrient substance, termed pigeon 'milk', for the nourishment of their young. Pigeons generally lay two eggs one day apart, which hatch 18 days after they are laid [1]. Two days before the first egg hatches, pigeon 'milk' begins to be produced in the crop of the parent birds. A similar substance is produced by flamingos [2] and male emperor penguins [3]. As in any other bird species, the normal function of the crop is as a food storage area located between the oesophagus and proventriculus where food is moistened before further break-down and digestion through the gastrointestinal tract [1]. During the process of pigeon 'lactation', a curd-like substance is regurgitated from the crop to the squab. Studies on pigeon 'milk' have shown that the dry matter is made up of 60% protein and the remainder is mostly fat (32-36%) and a small amount of carbohydrate (1-3%), in addition to the mineral (calcium, potassium, sodium, and phosphorus) content [4]. When this diet was artificially replicated and fed to squabs, their growth was either very poor or they died [5], which suggests there is a unique factor or factors present in pigeon 'milk' that is required for squab growth and development. Conversely, in a 1952 study where pigeon 'milk' was fed to chickens, their rate of growth improved by 38% [6]. Since this study, it has been shown that pigeon 'milk' contains IgA antibodies [7,8], which provides further evidence to suggest that it is more than a nutrient based substance.

The physiological mechanisms governing pigeon 'milk' production and delivery are unknown. It is well documented that the pigeon crop is responsive to the lactogenic hormone prolactin [9-11], however, histological studies on pigeon crop tissue during 'lactation' suggest that the process is structurally unrelated to traditional mammalian lactation because the pigeon crop is not glandular and secretory processes do not seem to be involved [12-14].

This study investigated the global gene expression profiles of pigeons that were 'lactating' as well as those that were not 'lactating' to identify genes that were differentially regulated in the pigeon crop during 'lactation'. Since the pigeon genome has not been sequenced and there are few gene sequences available in the public databases, we used a chicken microarray to probe gene expression in the pigeon crop. Publicly available pigeon genes have 98% nucleotide sequence identity to chicken (personal communication, A/Prof. Christophe Lefevre), and this chicken array has previously been shown to have utility in gene expression studies in other bird species [15].

## Results

### Macroscopic and histological comparison of 'lactating' and non- 'lactating' pigeon crop

**Figure 1:**
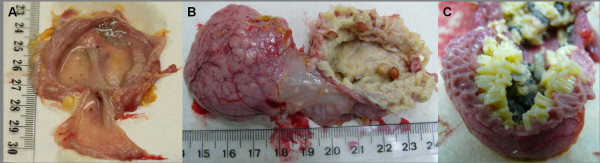
**Macroscopic appearance of the pigeon crop sac**. The non-'lactating' crop (A) has a completely different appearance to that of the 'lactating' crop (B). The lactating crop is more than twice the size of the non-'lactating' crop, with a thickened wall and two very obvious lateral lobes. When the 'lactating' crop is opened the pigeon 'milk' is seen as a bed of close-packed discrete rice-shaped pellets that are closely associated with the mucosal surface of the tissue (C). In contrast, the non-'lactating' crop is undifferentiated with minimal surrounding vasculature.

Macroscopic comparison of the 'lactating' and the non-'lactating' pigeon crop revealed two very different tissues (Figure 1). The pigeon crop lies between the distal oesophagus and the proximal end of the proventriculus. In its 'lactating' form, the crop is enlarged and has a thickened wall with two very obvious lateral lobes (Figure 1b). This contrasts with the non-'lactating' crop which is a thin-walled membranous sac (Figure 1a). When the 'lactating' crop is opened, the pigeon 'milk' appears as a bed of close-packed discrete rice-shaped pellets, each pellet being embedded in the mucosal surface of the lateral lobes (Figure 1c).

**Figure 2:**
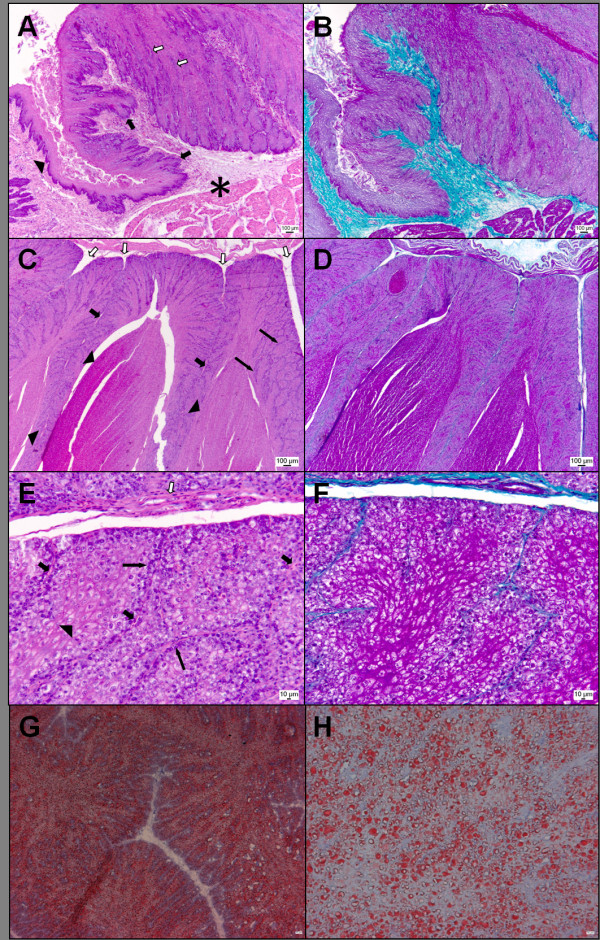
**Histological analysis of the pigeon crop sac**. (A) Junction of the non-'lactating' and 'lactating' walls of the lateral lobe of the pigeon crop. There is evidence of early hyperplasia in the non-'lactating' region (black arrowhead) with the formation of small rete pegs. These rete pegs are more pronounced as the hyperplasia becomes more obvious (black arrows), and proliferation of the germinal layer into the epithelium is also apparent (white arrows). *: lamina propria. Haematoxylin and eosin. (B) Same as A but stained with Masson's trichrome. The vascularized lamina propria is stained green. (C) 'Lactating' epithelium of the lateral lobe. There is extensive proliferation and folding of the basal layer of the epithelium. Branches of lamina propria extend into the epithelium (white arrows), and become progressively more attenuated (thick black arrows). These vascularized branches are narrowest near the luminal surface of the crop (thin black arrows). Pigeon 'milk' rice-shaped pellets, composed of lipid-laden, parakeratinised cells, are embedded closely in the epithelium (black arrowheads). Haematoxylin and eosin. (D) Same as C, but stained with Masson's trichrome. The green branches of the lamina propria are seen more readily with this stain. (E) High magnification of the epithelial wall of the lateral lobe of the lactating crop. Small vascularized branches of the lamina propria (thick black arrows) originate from a major branch (white arrow). One, two or three layers of slightly basophilic basal cells line these branches (thin black arrows), but, more removed from the blood supply, the cells have an eosinophilic, ground-glass appearance (black arrowhead). Haematoxylin and eosin. (F) Same as E, but stained with Masson's trichrome. The vascularized branches of the lamina propria can be seen quite clearly, as can the regions of protein-rich cells. (G) Lactating epithelium stained with Oil Red O (X5). Lipid can be seen consistently throughout the epithelium and is not present in the lamina propria or basement membrane. (H) Magnified view (X20) of lipid droplets in the epithelia of 'lactating' pigeon crop.

Histological examination of the wall of the lateral lobes in the 'lactating' crop (Figure 2) revealed a structure consistent with a number of previous detailed descriptions [12-14]. Moving from the non-lactating region of the crop into a lactating lobe, there was increasing papillary hyperplasia of the epithelium. This was characterized, firstly, by an increase in the number, and depth, of rete pegs extending from the germinal (basal) layer of the epithelium down into the lamina propria. As these rete pegs became deeper, there was also a concomitant proliferation, and very extensive upward folding, of the germinal layer into the superficial epithelium (Figure 2a, b). At all levels, every microscopic fold of the germinal layer invested a branch of vascularised lamina propria, although these branches became progressively narrower, and the associated blood vessels more attenuated, approaching the luminal surface of the crop wall (Figure 2c, d). Within the microscopic folds of epithelium, the germinal layer was mitotically active, and usually 1 to 3 cells thick whereas the overlying nutritive layer [12], also only 3 or 4 cells thick, had a pink, highly eosinophilic appearance, indicating high protein content of the differentiated cells (Figure 2e, f) and a ground-glass appearance indicative of hypoxia.

Near the luminal surface of the crop, the nutritive layers within each microscopic fold of epithelium began to coalesce, forming a parakeratinised layer. Macroscopic folds of the epithelium then resulted in fusion of large tracts of this parakeratinised layer, the end-result being the formation of discrete 'milk' pellets composed of avascular epithelium (Figure 2c, d). The cells within the pellets are nucleated, and are almost uniformly vacuolated. An Oil Red O stain revealed that the vacuoles contained lipid (Figure 2g, h).

### Cross-species microarray hybridisation profiling of the pigeon crop

A comparison of gene expression between four 'lactating' and four non-'lactating' female pigeon crops was made using pigeon cDNA hybridised to chicken long oligo-nucleotide microarrays [15]. When the data was subjected to Student's t-test using a significance value of *p *< 0.05, a list of 1181 genes were found to be differentially expressed between 'lactating' and non-'lactating'. Of these genes, 542 genes are up-regulated in the 'lactating' crop, and 639 genes are down-regulated. Of the genes up-regulated in 'lactating' crop, 113 are greater than 5-fold differentially expressed, and 407 are greater than 2-fold differentially expressed. Of the genes down-regulated in 'lactating' crop, 171 are more than 5-fold differentially expressed and 608 are greater than 2-fold differentially expressed. The data has been deposited into the public database ArrayExpress (accession: E-MEXP-3314).

### Functional analysis of gene expression in the 'lactating' pigeon crop sac

Pathway analysis of differentially expressed genes revealed four pathways enriched in 'lactating' crop tissue; melanogenesis, extracellular matrix (ECM)-receptor interaction, adherens junction and the wingless (wnt) signalling pathway (Table 1). The most enriched pathway, melanogenesis, represents 2.5% of all up-regulated genes, closely followed by the wnt signalling pathway, representing 2.2% of all up-regulated genes. The genes involved in the melanogenesis pathway range from 1.5-fold to 5.9-fold up-regulated in 'lactating' crop, the highest fold-change being that of the transcription factor, *micropthalmia-associated transcription factor *(*MITF*). Histological examination of the crop (Figure 2) failed to identify the presence of pigmented melanosomes, which are the products of melanogenesis.

**Table 1:** Enriched KEGG pathways in 'lactating' crop transcriptome

Pathway	% Total genes^	%Mapped genes*	*p *value
Melanogenesis	2.5	10.5	2.7e-3
ECM-receptor interaction	1.9	7.9	2.2e-2
Adherens junction	1.6	6.6	6.3e-2
Wnt signalling pathway	2.2	9.2	6.6e-2

Gene expression pathways that were identified as enriched in 'lactating' crop tissue.

^Total genes is the number of up-regulated genes (320) that could be identified by the DAVID program

*Mapped genes is the number of genes (76) that could be mapped to a KEGG pathway.

**Figure 3:**
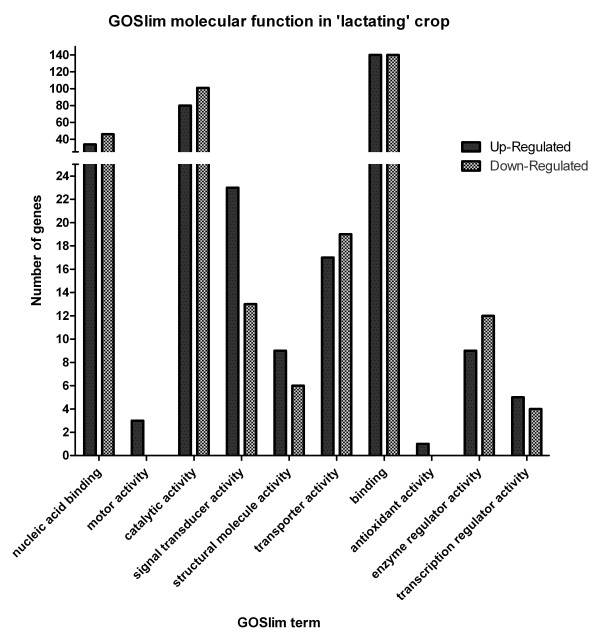
**Gene ontology molecular function of genes differentially expressed in the 'lactating' pigeon crop sac**. Differentially expressed genes that could be assigned a UniGene ID (496) were matched to any gene ontology GO Slim ID in the database. The number of genes in each molecular function is presented here. Genes with the molecular functions motor activity and antioxidant activity are present only in 'lactating' crop.

Gene ontology analysis of differentially expressed genes revealed two molecular functions that have genes up-regulated only in the 'lactating' crop; those functions being motor activity and antioxidant activity (Figure 3). Inspection of the two genes with the molecular function motor activity reveals they are both genes encoding cytoplasmic dynein proteins (Table 2), which are involved in cellular transport via microtubules. *Peroxiredoxin 1 *represents the molecular function antioxidant activity, and is highly up-regulated in 'lactating' crop by 21-fold. Further investigation identified three genes encoding heat shock proteins (Additional file 1) which can also exert similar effects as antioxidant proteins. Analysis of genes annotated by gene ontology as having an immune function revealed three up-regulated genes that are platelet factors/mediators; *CXCL4 *(*platelet factor 4*), *CD36 *and *coagulation factor VII *(Additional file 2).

**Table 2:** Genes with a molecular function that is present only in 'lactating' crop sac

UniGene ID	Gene name	Fold change	*p *value
**Motor activity (GO:0003774)**			
Gga.26348	Dynein, cytoplasmic 2, heavy chain 1	2.3	0.0277
Gga.25866	Dynein, cytoplasmic 1, light intermediate chain 1	2	0.0291
**Antioxidant activity (GO:0016209)**			
Gga.5204	Peroxiredoxin 1	19	0.00231

Fold change and significance of genes that have a gene ontology molecular function that is present only in 'lactating' crop tissue.

## Discussion

The 'lactating' pigeon crop has a distinctly different appearance to the non-'lactating' crop that is reflected both macroscopically (Figure 1) and histologically (Figure 2). Global gene expression profiling of 'lactating' and non-'lactating' pigeon crop showed that gene expression patterns were also distinctly different in the 'lactating' crop as compared to the non-'lactating' crop.

The proliferation of the crop germinal epithelium (Figure 2) may be induced by the transcriptional stimulatory activity of *micropthalmia-associated transcription factor *(*MITF*) which is up-regulated in 'lactating' crop by 5.9-fold. This is a key gene in the melanogenesis pathway [16], which is enriched in 'lactating' crop (Table 3). There are two different signalling pathways that can participate in the signalling cascade that results in proliferation of melanocytes through the melanogenesis pathway; the mitogen activated protein kinase (MAPK) signalling pathway [17] and the wingless (wnt) signalling pathway [16]. The wnt signalling pathway is enriched in 'lactating' crop (Table 1) whereby seven genes are up-regulated, including the wnt signalling receptor, *Frizzled homolog 3*, by 2.8-fold and *beta-catenin*, a mediator of *MITF *signalling [18], by 2.1-fold. As there is no evidence of melanocytes in the pigeon crop, in either its 'lactating' or 'resting' state, we propose that either *MITF *targets a different cell type in the pigeon crop or that the *MITF *probe on our chicken microarray is similar to another transcription factor in the pigeon that is important in inducing the proliferative signalling pathway during 'lactation'. These signalling pathways may act on a stem cell population that gives rise to the proliferative germinal epithelium which produces the cells that eventually form the tracts of parakeratinised cells termed pigeon 'milk' (Figure 2) through extracellular matrix-receptor interactions. This pathway is enriched in 'lactating' crop (Table 1), with 6 genes up-regulated (Table 3).

**Table 3:** Fold change and significance of genes involved in enriched KEGG pathways

UniGene ID	Gene name	Fold change	*p *value
**Melanogenesis pathway**			
Gga.275	similar to Mitf; microphthalmia-associated transcription factor	5.9	0.0161
Gga.3044	wingless-type MMTV integration site family, member 6	3.5	0.0392
Gga.606	v-kit Hardy-Zuckerman 4 feline sarcoma viral oncogene homolog	2.8	0.0252
Gga.3839	frizzled homolog 3 (Drosophila)	2.8	0.0354
Gga.7247	cAMP responsive element binding protein 3-like 3	2.4	0.0249
Gga.5777	cAMP responsive element binding protein 3	2.2	0.0131
Gga.4283	catenin (cadherin-associated protein), beta 1, 88 kDa	2.2	0.00158
Gga.42842	v-raf-1 murine leukemia viral oncogene homolog 1	1.5	0.032
**ECM-receptor interaction**			
Gga.13583	similar to fatty acid translocase; similar to CD36 molecule (thrombospondin receptor)	5.3	0.0142
Gga.1784	integrin, alpha 8	2.2	0.0492
Gga.40239	synaptic vesicle glycoprotein 2C	2	0.0409
Gga.15246	collagen, type V, alpha 2	2	0.00304
Gga.42140	collagen, type III, alpha 1	2	0.0224
Gga.9475	dystroglycan	1.9	0.0375
**Adherens junction**			
Gga.30780	activin A receptor, type IB	14.2	0.0209
Gga.3243	casein kinase 2, alpha 1 polypeptide	2.8	0.0495
Gga.4404	protein tyrosine phosphatase, non-receptor type 1	2.7	0.0173
Gga.4283	catenin (cadherin-associated protein), beta 1, 88 kDa	2.2	0.00158
Gga.12594	similar to TGF-beta type II receptor	1.8	0.0247
**Wnt signalling pathway**			
Gga.10637	vang-like 2 (van gogh, Drosophila)	39.6	0.028
Gga.8363	axin 2	3.6	0.0268
Gga.3044	wingless-type MMTV integration site family, member 6	3.5	0.0392
Gga.3243	casein kinase 2, alpha 1 polypeptide	2.8	0.0495
Gga.3839	frizzled homolog 3 (Drosophila)	2.8	0.0354
Gga.4283	catenin (cadherin-associated protein), beta 1, 88 kDa	2.2	0.00158
Gga.1473	jun oncogene	2.1	0.0293

Genes that are differentially expressed between 'lactating' and non-'lactating' crop and play a role in the KEGG pathways that were identified as enriched in 'lactating' crop tissue.

There are areas of cells in the 'lactating' crop that have an altered, ground-glass appearance (Figure 2e, f). We speculate that this is a result of hypoxia caused by lack of blood supply to the rapidly proliferating germinal cell layer of the pigeon crop, in addition to the oxidative by-products of this rapid proliferation. *Peroxiredoxin 1*, a major cytosolic antioxidant protein encoding gene involved in cell redox homeostasis is up-regulated by more than 21-fold in the 'lactating' crop (Table 2). Other markers of cellular stress include the *heat shock proteins (hsp) *family which proliferate in response to multiple types of cellular stress, including inflammation and hypoxia [19]. Three of these *hsp *genes (Additional file 1) are up-regulated in the 'lactating' crop by between 1.8 and 7.7-fold. As well as their role in stress response, they are likely to play an important role in protein folding and transport [20] in the 'lactating' crop, which produces 'milk' containing a high proportion of protein.

There are multiple immune genes up-regulated in the tissue (Table 4), including the gene encoding the chemokine *CXCL4 *or *platelet factor 4 *(1.6-fold up-regulated), a gene expressed by platelets [21], which suggests that there is platelet infiltration in the tissue. CD36 (5.3-fold up-regulated), a membrane glycoprotein, can function as a platelet adhesion mediator [22], and coagulation factor VII (1.8-fold up-regulated) is part of the clotting pathway stimulated by tissue damage [23]. It is possible that if antioxidant and immune proteins are present in pigeon 'milk', they are directly enhancing the immune system of the developing squab as well as protecting the parental crop tissue. Moreover, the gene encoding the somatostatin receptor, which has been shown to modulate intestinal activity and inhibit nutrient resorption during periods of inflammation [24], is up-regulated in the pigeon crop during 'lactation' by more than 2-fold (Additional file 1). This is not surprising, given that the pigeon crop is part of the gastrointestinal tract. A clear physiological change in the 'lactating' crop is that of lipid accumulation (Figure 2g, h). Dumont (1965) showed that this lipid is neutral unsaturated triglyceride, that it is present both in the pigeon crop and the pigeon 'milk', and has a nutritive function. Avian keratinocytes, in contrast to mammalian keratinocytes, can accumulate lipid during cornification of the epidermis [25]. It appears that in the evolution of the mechanism pigeon 'milk' production process, the pigeon crop has extended on the ability of keratinocytes to accumulate nutritive lipids, to produce a cellular substance for the nourishment of their young. During the process of pigeon 'milk' synthesis, the crop sac produces copious amounts of epidermis, composed of parakeratinised cells, which become cornified toward the luminal surface of the tissue (Figure 2). We found that there is an up-regulation of two *Acyl-CoA synthetase *genes (1.8 and 2.5-fold) in 'lactating' crop tissue. These genes encode enzymes that are part of the fatty acid oxidation pathway that precedes the synthesis of triglycerides in the cell [26]. The fatty acid precursors of these triglycerides are likely to be obtained through the blood supply from oxidised triglycerides of the adipose tissue or liver. This is in agreement with Garrison and Scow (1975) who proposed that crop triglyceride is sequestered from another organ through the blood supply [27]. Oxidised triglyceride is transported from the liver or adipose tissue as fatty acids on very-low density lipoproteins (VLDL) that enter the cell by endocytosis [26]. KEGG pathway analysis revealed that the endocytosis pathway is enriched in 'lactating' crop (Table 1). Once triglycerides are synthesised in the cytoplasm of the cell, they are packaged into vesicles; the up-regulation of cytoplasmic dynein in 'lactating' crop (dynein cytoplasmic 1, light intermediate chain 1; 1.9-fold, and dynein cytoplasmic 2, heavy chain 1; 2.5-fold) suggests that pigeon crop triglyceride is transported via the microtubules to vacuoles within the cell (Figure 2g, h).

**Table 4:** Genes differentially expressed between 'lactating' and non-'lactating' pigeon crop that have an immune function

UniGene ID	Gene name	Fold change	*p *value
Gga.22171	Similar to Toll-like receptor 21	10.6	0.0269
Gga.13583	CD36 molecule (thrombospondin receptor)	5.3	0.0142
Gga.686	Bone morphogenetic protein 4	4.6	0.00317
Gga.4852	Cytokine receptor-like factor 3	3.1	0.0267
Gga.21395	Thymocyte selection-associated high mobility group box	3.1	0.022
Gga.25682	Serologically defined colon cancer antigen 8	2.4	0.000345
Gga.5132	Coagulation factor VII (serum prothrombin conversion accelerator) (F7)	1.9	0.0112
Gga.1239	Mitochondria-associated protein involved in granulocyte-macrophage colony-stimulating factor signal transduction	1.8	0.0444
Gga.4409	Chemokine (C-C motif) ligand 4	1.7	0.0239
Gga.1020	Chemokine (C-C motif) ligand 17	0.4	0.0453
Gga.296	Chemokine (C-C motif) ligand 1	0.4	0.0176
Gga.40421	Hypothetical LOC420181	0.4	0.0206
Gga.22379	Receptor-interacting serine-threonine kinase 2	0.4	0.00553
Gga.21994	Immunoresponsive 1 homolog (mouse)	0.4	0.0198
Gga.11155	Stromal antigen 2	0.4	0.0233
Gga.24900	Interleukin 18 receptor 1	0.3	0.00839
Gga.5128	Chemokine (C-C motif) ligand 20	0.2	0.00172
Gga.1913	Replication factor C (activator 1) 1, 145 kDa	0.2	0.0301
Gga.39923	Similar to chronic myelogenous leukemia tumor antigen 66	0.2	0.0157
Gga.7865	Interleukin-1 receptor-associated kinase 2	0.1	0.044
Gga.23148	Recombination activating gene 2	0.08	0.0458
Gga.34578	Similar to suppressor of cytokine signalling 1	0.07	0.0342
Gga.46998	Suppressor of cytokine signalling 7	0.04	0.0449

Fold change and significance of genes that have an immune function.

This study has provided a snap-shot view of some of the processes occurring when 'lactation' in the pigeon crop is well established. Due to the unusual nature of 'lactation' in the pigeon it would be interesting to investigate the early stages of the differentiation and development of the crop in preparation for 'milk' production to further ascertain gene expression patterns that characterise crop development and 'lactation' in the pigeon. The use of pigeon-specific genomic or transcriptomic material would allow the identification of genes that are unique to the pigeon or that are dissimilar enough to the chicken to elude identification using a cross-species method.

## Conclusions

The study of gene expression in the 'lactating' crop is starting to shed light on the biological processes occurring during this unusual biological process. The evolution of pigeon crop 'milk' production appears to have developed from the ability of avian keratinocytes to accumulate lipid. The picture that emerges from an analysis of gene expression changes in the crop is of a process that is characterised by a hyperplastic response in the pigeon crop epithelium which results in the accumulation of lipid-containing, cornified keratinocytes in the crop lumen. This leads to localised cellular stress and subsequent expression of antioxidant protein-encoding genes including *peroxiredoxin 1*. This hypothesised functional explanation is consistent with the histological examination of the 'lactating' crop presented here and in other studies.

## Methods

### Sample collection

Eight pairs of King pigeons were purchased from Kooyong Squab Producers (Moama, New South Wales). They were housed in temperature-controlled cabinets (between 21-24°C) with a 12 hour light cycle (lights on 6 am), and supplied with nest bowls and nesting materials. Pigeons had ad lib access to pigeon mix (pro-vit-min, Ivorsons, Geelong) and water. Four pairs were culled prior to mating, making up the non-'lactating' group, and the other four were culled 48 hours post-hatch of the first squab; the 'lactating' group. Crop tissue samples were snap frozen in liquid nitrogen and separate samples were fixed in 10% neutral buffered formalin for histology.

All work using animals was conducted in accordance with the Australian Code of Practice for the Care and Use of Animals for Scientific Purposes (7^th ^edition), and in accordance with institutional animal ethics guidelines (CSIRO AAHL Animal Ethics Committee).

### RNA isolation, labelling and microarray hybridisation

Total RNA was isolated from the crop tissue of four female 'lactating' pigeons and four female non-'lactating' pigeons using TriReagant RT (Molecular Research Centre) with an additional high salt solution step to precipitate glycoproteins. cDNA labelled with Cy3 (Kreatech) was synthesised from 5 μg of total RNA using SuperScript III (Invitrogen) with oligo(dT)_20 _primer and concentrated to less than 4 μl using a microcon YM-30 column (Millipore). Custom 20 k chicken long oligonucleotide microarrays [15] were blocked with 5 mL pre-hybridisation solution (25% Formamide, 5XSSC, 1% SDS, 1% BSA, 0.1% salmon sperm DNA) at 42°C for one hour in an incubation chamber, rinsed with double distilled water and dried in a centrifuge (5 minutes at 195 × *g*). Pre-hybridised microarrays were hybridised with hybridisation buffer containing the labelled cDNA probe (25% Formamide, 5XSSC, 0.6% SDS, 25% kreablock to 30 μl) for 16 hours at 42°C in a water bath. They were then washed with gentle agitation in 2XSSC, 0.1% SDS for 1 minute, 0.2XSSC, 0.1% SDS for 10 minutes and twice in 0.1XSSC for 1 minute. After drying the slides by centrifugation (5 minutes at 195 × *g*) they were scanned at three exposure times (0.05, 0.10, and 0.15 seconds) using an arrayWoRx scanner (Applied Precision) and aligned to the microarray template to correlate each spot to an oligo-nucleotide identifier, using softWoRx tracker (Applied Precision).

### Quality control and statistical analysis

The most appropriate scanning exposure time was selected by choosing the datasets with the least number of saturated spots (scanning intensity > 65,000) and the highest number of spots above the average background (cell method of background calculation). The datasets were then normalised by multiplying the spot actual intensity (spot median intensity minus spot background intensity) by a nominal value of one million and dividing by the sum of all spot actual intensities. The datasets were exported into GeneSpring (Silicon Genetics) for statistical analysis and further normalised to the spot median intensity. A list of oligonucleotides that had a significantly different level of expression between conditions was obtained by applying a Student's t-test assuming unequal variances with a false discovery rate of *p *= 0.05.

#### Gene functional categorisation and pathways analysis

GO annotations were assigned to the microarray probes via UniGene IDs. The probe sequences were matched with the chicken UniGenes Build #41 downloaded from NCBI ftp://ftp.ncbi.nih.gov/repository/UniGene/Gallus_gallus/ using MegaBlast v2.2.21 [28]. The matching UniGene IDs were then used to link the probes with GO annotations and GOslim terms from the gene association and protein cross-references files for chicken, release 3.56 http://www.ebi.ac.uk/GOA/chicken_release.html This resulted in GO annotations for 41% of the probes on the microarray. The number of genes up or down-regulated in each GOSlim molecular function category was determined for each differentially expressed gene with a GO annotation, and graphed using GraphPad Prism 5 (GraphPad Software Inc., USA).

To identify pathways of interest important to pigeon 'lactation', only up-regulated genes were further investigated using the DAVID functional annotation tool [29]. It was used to determine which Kyoto Encyclopedia of Genes and Genomes (KEGG) pathways were enriched during pigeon 'lactation' using an ease score of 0.1. UniGene gene identifiers were converted to DAVID identifiers using the DAVID Gene ID conversion tool.

#### Histological examination

Tissues were fixed in 10% neutral buffered formalin and embedded in paraffin wax. Sections of 4 μm were cut, dewaxed with xylene, rehydrated through a series of ethanol washes and stained with either haematoxylin and eosin or Masson's trichrome. Masson's trichrome stained muscle red and collagen blue.

Separate pieces of tissue were snap frozen in OCT and sections of 8 μm were cut with a cryostat, stained with oil red O and counterstained with haematoxylin as per the method of [30].

## Authors' contributions

TC and RM conceived the project and MG, JD, KN and PM contributed to formulation of ideas. MG did the experimental work and wrote the manuscript. KM analysed the histology slides. VH carried out Gene Ontology analysis. All authors read and approved the final manuscript.

## Supplementary Material

Additional file 1 **List of all genes differentially expressed between 'lactating' and non-'lactating' crop tissue**. This table contains all of the microarray probes that are differentially expressed between 'lactating' and non-'lactating' crop. The probe identifier, probe sequence, fold-change, p value and annotation (if any) are given for each probe in the table. Click here for file

Additional file 2 **List of all genes assigned a molecular function**. This table contains all of the differentially expressed genes that could be assigned a gene ontology molecular function using UniGene IDs. The GO Slim identifier, UniGene ID, and GO ID are given for each gene in the table. Click here for file
